# The Anticancer Agent Elesclomol Has Direct Effects on Mitochondrial Bioenergetic Function in Isolated Mammalian Mitochondria

**DOI:** 10.3390/biom9080298

**Published:** 2019-07-24

**Authors:** Josephine S. Modica-Napolitano, Leena P. Bharath, Alison J. Hanlon, Liam D. Hurley

**Affiliations:** 1 Department of Biology, Merrimack College, North Andover, MA 01845, USA; 2 Department of Health Sciences, Merrimack College, North Andover, MA 01845, USA

**Keywords:** mitochondria, bioenergetics, elesclomol, anti-cancer

## Abstract

Elesclomol ((*N*-malonyl-bis(*N′*-methyl-*N′*-thiobenzoylhydrazide)); formerly STA-4783) is a mitochondria-targeted chemotherapeutic agent that has demonstrated efficacy in selective cancer cell killing in pre-clinical and clinical testing. The biologically active form of elesclomol is a deprotonated copper chelate (elesclomol:copper; E:C), which has been shown to enhance reactive oxygen species (ROS) production and induce a transcriptional gene profile characteristic of an oxidative stress response in vitro. Previous studies suggest that E:C interacts with the electron transport chain (ETC) to generate high levels of ROS within the organelle and ultimately induce cell death. The purpose of this study was to further explore the mechanism of cellular and mitochondrial toxicity of E:C by examining its direct effect on mitochondrial bioenergetic function. The results obtained indicate that E:C treatment in whole cells of non-tumorigenic origin at high concentrations (40 μM and higher) induces a rapid and substantial increase in mitochondrial superoxide levels and dissipation of mitochondrial membrane potential. Furthermore, similar higher concentrations of E:C act as a direct uncoupler of oxidative phosphorylation and generalized inhibitor of electron transport activity in isolated, intact mitochondria, and induce a dose-dependent inhibition of mitochondrial NADH-ubiquinone oxidoreductase activity in freeze-thawed mitochondrial preparations. The results of this study are important in that they are the first to demonstrate a direct effect of the E:C chelate on bioenergetic function in isolated mammalian mitochondria, and suggest the possibility that the increase in ROS production and cytotoxicity induced by E:C may in part be due to uncoupling of mitochondrial oxidative phosphorylation and/or inhibition of electron transport activity. These results also provide important information about the mechanisms of mitochondrial and cellular toxicity induced by E:C and will ultimately contribute to a better understanding of the therapeutic potential of elesclomol as an anticancer compound.

## 1. Introduction 

**Figure 1:**
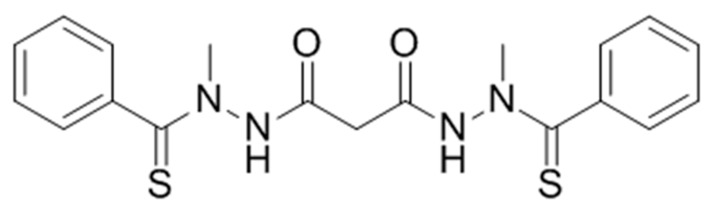
Chemical structure of elesclomol (MedChemExpress, https://www.medchemexpress.com/).

Elesclomol (*N*-malonyl-bis(*N′*-methyl-*N′*-thiobenzoylhydrazide)); formerly STA-4783; Figure 1) is a mitochondria-targeted chemotherapeutic agent that has exhibited antitumor activity against a broad range of cancer cell types in vitro, enhanced the potency of known chemotherapeutic agents in human tumor models in vivo, and displayed encouraging but limited therapeutic benefit as a single agent and in combination with other anti-cancer compounds in Phase I through Phase III clinical trials [1,2,3,4,5,6]. 

Elesclomol has been shown to induce a rapid accumulation of intracellular reactive oxygen species (ROS) and a gene transcription profile characteristic of an oxidative stress response in vitro [7]. Interestingly, the antioxidant *N*-acetylcysteine blocks elesclomol induced gene expression and cell death, suggesting that ROS generation may be the primary means of cancer cell killing by the drug [7]. 

The biologically active form of elesclomol is a deprotonated copper chelate [8]. Upon therapeutic administration, this chelate forms when elesclomol acquires Cu^2+^ in the bloodstream. However, in vitro studies investigating the mechanism of action of elesclomol have demonstrated that the addition of a pre-formed elesclomol:copper (E:C) complex is necessary to induce cytotoxicity [8]. In one such in vitro study, comparative growth assays using deletion mutants of a yeast model yield evidence that E:C works through a biologically coherent set of processes occurring in the mitochondrion [9]. The results suggest that E:C interacts with the electron transport chain (ETC), a major component of the process of oxidative phosphorylation, to generate high levels of ROS within the organelle and ultimately induce cell death. The authors concluded that E:C mediated cytotoxicity occurs via general disruption of the process of electron flow down the ETC, rather than by targeting any particular protein or enzyme function within the ETC. 

The purpose of this study was to further explore the mechanism of cellular and mitochondrial toxicity of E:C by examining its direct effect on mitochondrial bioenergetic function in whole cells of non-tumorigenic origin and in isolated mammalian mitochondria. The results obtained indicate that E:C treatment in whole cells induces a rapid and substantial increase in mitochondrial superoxide levels and dissipation of mitochondrial membrane potential. Furthermore, E:C acts as an uncoupler of oxidative phosphorylation and generalized inhibitor of electron transport activity in isolated, intact mitochondria, and induces a dose-dependent inhibition of mitochondrial NADH-ubiquinone oxidoreductase activity in freeze-thawed mitochondrial preparations. The results of this study are important in that they are the first to demonstrate a direct effect of the E:C chelate on bioenergetic function in isolated mammalian mitochondria, and suggest the possibility that the increase in ROS production and cytotoxicity induced by E:C may in part be due to uncoupling of mitochondrial oxidative phosphorylation or inhibition of electron transport activity, or both.

## 2. Materials and Methods

### 2.1. Materials 

Elesclomol was obtained from MedChemExpress (https://www.medchemexpress.com/) and made fresh daily at a concentration ranging from 1–10 mM in dimethyl sulfoxide (DMSO). The E:C complex was formed by mixing equimolar and equivolume additions of elesclomol and copper chloride (dissolved in H_2_O). MitoSOX Red mitochondrial superoxide indicator for live-cell imaging was obtained from Molecular Probes (Eugene, OR, USA); the mitochondrial superoxide scavenger MitoTEMPO was obtained from Sigma Aldrich (St. Louis, MO, USA); the mitochondrial membrane potential probe TMRE (tetramethylrhodamine, ethyl ester) was obtained from Biotium (Fremont, CA, USA); and the live cell nuclear stain, NucBlue Live, and the cell viability stain, NucGreen Dead, were obtained from ThermoFisher Scientific (Waltham, MA, USA). All solutions were used according to the manufacturer’s specifications.

### 2.2. Cell Cultures 

The CV-1 African green monkey kidney epithelial cells (ATCC^®^ CCL-70^™^) were grown in Eagle′s minimal essential medium (EMEM) supplemented with 10% fetal bovine serum. All cells were maintained at 37 °C in a 5% CO_2_ atmosphere.

### 2.3. Mitochondrial Superoxide Production in Whole Cells

Mitochondrial superoxide production was assessed by measuring MitoSOX fluorescence using a Zeiss 800 confocal microscope (Carl Zeiss Microscopy GmbH, Jena, Germany). The CV-1 cells were seeded in glass bottom 35 mm plates (MatTek) and grown to 90% confluency. To initiate an experiment, cells were incubated for 10 min with 5 μM MitoSOX in phenol red free EMEM, washed twice with sterile phosphate buffered saline, and replenished with phenol red free EMEM and 2 drops/mL media of NucBlue or NucGreen. Cells were then treated with either DMSO (control) or 70 μM E:C, and MitoSOX, NucBlue, and/or NucGreen fluorescence was monitored over a period of 1 h. To block mitochondrial superoxide accumulation, some cells underwent a 1-h pre-treatment with 5 μM of the free radical scavenger MitoTEMPO prior to addition of DMSO or E:C. 

### 2.4. Mitochondrial Membrane Potential in Whole Cells

Mitochondrial membrane potential was assessed by measuring TMRE fluorescence using a Zeiss 800 confocal microscope. The CV-1 cells were seeded in glass bottom 35 mm plates (MatTek) and grown to 90% confluency. To initiate an experiment, cells were washed twice with sterile phosphate buffered saline and incubated for 10 min with 100 nM TMRE in phenol red free EMEM and two drops/ml media of NucBlue or NucGreen. Cells were then treated with either DMSO (control) or 70 μM E:C, and TMRE and NucBlue and/or NucGreen fluorescence was monitored over a period of 1 h. As a positive control for uncoupling of oxidative phosphorylation and dissipation of mitochondrial membrane potential, cells were incubated for 10 min in the presence of the ionophore FCCP (carbonyl cyanide-*4*-(trifluoromethoxy)phenylhydrazone; 100 μM). 

### 2.5. Isolation of Mitochondria 

Mitochondria were isolated from bovine or rat liver by a process of differential centrifugation at 4 °C [10]. Briefly, 5–6 g of tissue were minced and homogenized in STE (250 mM sucrose, 1 mM Tris-HCI, and 1 mM EDTA (ethylenediaminetetraacetic acid, pH 7.4)), 20% *w/v*, and centrifuged at 600× *g* for 10 min. The supernatant was collected and then centrifuged for 10 min at 8000× *g*. The resulting mitochondrial pellet was re-suspended and washed twice by centrifugation for 10 min at 8000× *g* in STE, followed by an additional wash in ST (250 mM sucrose and 1 mM Tris-HC1 (pH 7.4)). The final pellet was re-suspended in ST and the protein concentration of the mitochondrial suspension was determined by the method of Lowry [11].

### 2.6. Mitochondrial Respiration 

Oxygen consumption was measured in freshly isolated rat liver mitochondria using a Clark-type polarographic oxygen electrode (Oxytherm, Hansatech Instruments, PP Systems Inc., Amesbury, MA, USA) in a solid-state Peltier temperature controlled chamber maintained at 30 °C. The basic respiratory assay medium consisted of 225 mM sucrose, 10 mM KCl, 1 mg/mL 10 mM K_2_HPO_4_-KH_2_PO_4_, 5 mM MgCl_2_, and 10 mM Tris-HCl (pH 7.4). An initial rate of oxygen consumption (state 2 rate) was recorded after the addition of mitochondria (approximately 1.5 mg) and respiratory substrate (5 mM each glutamate plus malate or 10 mM succinate). The ADP (120 nmol) was then added to obtain a state 3 rate, which is a measure of oxygen consumption that is coupled to the process of ADP phosphorylation to ATP. Following a measurable state 4 rate (i.e., the respiratory rate after all added ADP has been phosphorylated), 80 µM of the chemical uncoupler 2,4-dinitrophenol was added to obtain a respiratory rate in the absence of coupled oxidative phosphorylation. Respiratory rates were measured in the absence or presence of varying concentrations of E:C (25–100 μM).

### 2.7. Enzyme Assays 

Spectrophotometric measurements of mitochondrial electron transport enzyme activities were obtained using a single-wavelength, temperature-controlled spectrophotometer at 37 °C essentially as described previously [10,12]. All enzyme activities were assayed in a final reaction volume of 1.0 mL. As elesclomol, which is dissolved in DMSO, was varied in the assays, additional DMSO was added as necessary to maintain a constant final volume of the solvent in each reaction mixture. For each assay, triplicate values of enzyme activity were obtained for control runs and at every concentration of E:C tested.

The NADH-ubiquinone oxidoreductase activity (a measure of electron transport through respiratory complex I) was determined by measuring the rate of decrease in absorbance at 340 nm due to the oxidation of NADH. Mitochondria (300 µg) that had been subjected to three cycles of freeze-thawing in liquid nitrogen were added to a cuvette containing 50 mM potassium phosphate, 3 mg/mL fatty acid free BSA, 300 μM KCN, 100 μM NADH in the absence or presence of varying concentrations of E:C (25–100 μM). The reaction was initiated by adding 10 μM ubiquinone_1_ to the cuvette, and the change in the absorbance was recorded over time in the presence and absence of 10 μM rotenone. The rotenone sensitive activity of complex I of the mitochondrial ETC was determined by subtracting the rate measured in the presence of rotenone from the rate obtained in the absence of rotenone. 

Succinate-cytochrome c reductase activity (a measure of electron transport through respiratory complexes II and III) was determined by measuring the rate of increase in absorbance at 550 nm due to the reduction of oxidized cytochrome c. Freeze-thawed (−20 °C) preparations of isolated mitochondria (300 μg) were added to a cuvette containing dH_2_O, 50 mM potassium phosphate (KH_2_P0_4_/K_2_HP0_4_; pH 7.4), 30 μM KCN, in the absence or presence of varying concentrations of E:C (25–100 μM). The sample was pre-incubated with the 20 mM succinate for 10 min at 37 °C in order to fully activate the enzyme. The reaction was initiated by adding 50 μM oxidized cytochrome c to the cuvette, and a change in the absorbance was recorded over time. 

Cytochrome c oxidase activity (a measure of electron transport through complex IV) was determined by measuring the decrease in absorbance at 550 nm due to the oxidation of reduced cytochrome c. Freeze-thawed (−20 °C) preparations of isolated mitochondria (300 μg) were added to a cuvette containing dH_2_O, 40 mM potassium phosphate (KH_2_PO_4_/K_2_HPO_4_; pH 7.0), in the absence or presence of varying concentrations of E:C (25–100 μM). The reaction was initiated by adding 50 μM reduced cytochrome c, and a linear rate was recorded. 

## 3. Results

### 3.1. E:C-Induced Effects on Mitochondrial Superoxide Production and Membrane Potential

**Figure 2:**
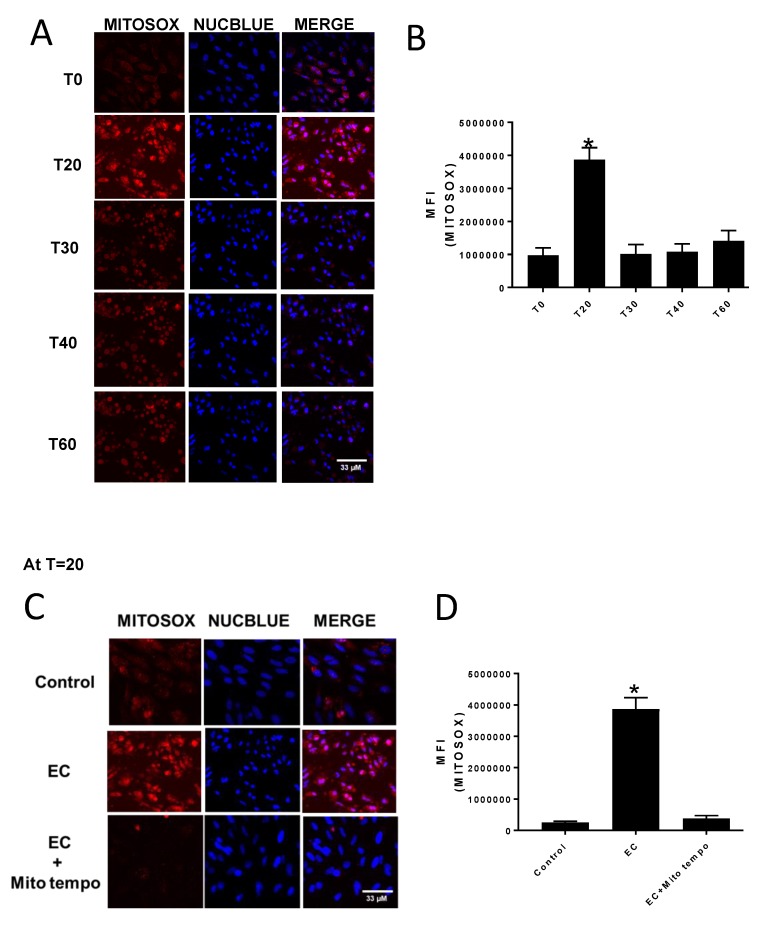
Monitoring mitochondrial superoxide production in whole cells. **(A**) Confocal images of control and elesclomol:copper (E:C)-treated (70 μM) CV-1 cells at various time points after staining with the mitochondria-specific superoxide indicator MitoSOX and the nuclear stain NucBlue Live. (**B**) Quantification of MitoSOX fluorescence intensity in CV-1 cells at the different time points after E:C treatment. (**C**) Confocal images of MitoSOX fluorescence intensity in CV-1 cells at the 20-min time point after E:C treatment in the presence and absence of the mitochondrial superoxide scavenger MitoTEMPO. **(D**) Quantification of MitoSOX fluorescence intensity in CV-1 cells at the 20-min time point after E:C treatment in the presence and absence of the mitochondrial superoxide scavenger MitoTEMPO. Images in the NucBlue Live channel were adjusted to improve visual clarity. This adjustment does not affect the quantification. A minimum of three cells/field and three fields/slide were imaged for each plate at 20× magnification using a Zeiss confocal microscope. The images were analyzed using Image J software, and the change in mean fluorescence among groups was plotted. Normality of distribution of the data was determined using the D-Agostino Omnibus test. A one-way ANOVA analysis was performed followed by a Dunn’s post hoc test using GraphPad Prism software (San Diego, CA, USA) to identify statistical difference among the samples. *N* = 3 for each condition; *N* represents one glass-bottomed 35 mm petri dish. * *p* < 0.05 vs control or T0.

The effect of E:C treatment on mitochondrial superoxide production in whole cells was determined using the live-cell permeant superoxide indicator, MitoSOX, which is selectively targeted to the mitochondria and fluoresces red upon oxidization by superoxide (but not by other ROS or reactive nitrogen species). Figure 2A,B shows that after 20 min, CV-1 cells treated with 70 μM E:C exhibited a 4-fold increase in superoxide generation. However, by 30 min (and beyond) the superoxide levels were comparable to the control value. Therefore, it is possible that the decrease in MitoSOX fluorescence observed over time may be due to a disruption of mitochondrial membrane integrity and loss of the dye. As expected, when CV-1 cells were treated with 70 μM E:C and MitoSOX in the presence of the mitochondria-targeted antioxidant MitoTEMPO, no increase in fluorescence was detected (Figure 2C,D), confirming that the observed increase in fluorescence with E:C treatment was due to an increase in mitochondrial superoxide production. 

**Figure 3:**
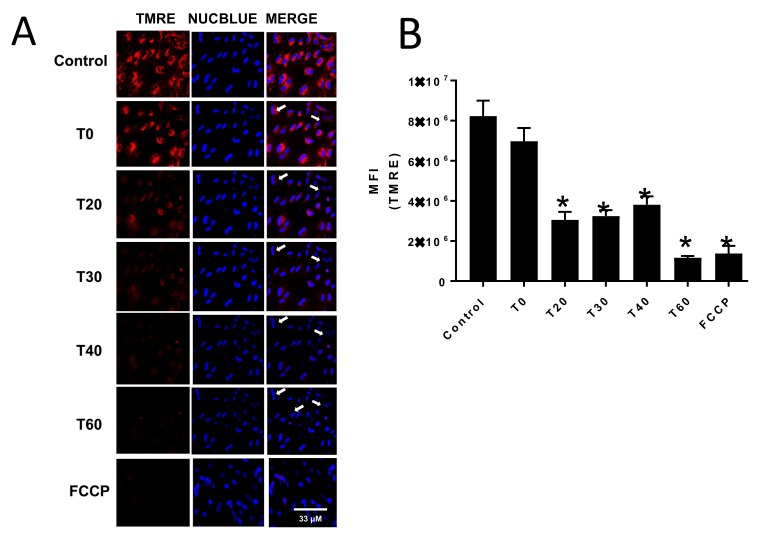
Monitoring the effect of E:Con mitochondrial membrane potential in whole cells at various time points. (**A**) Confocal images of control and E:C-treated (70 μM) CV-1 were observed at various time points after staining with the membrane potential dependent fluorescent dye tetramethylrhodamine, ethyl ester (TMRE) and the nuclear stain NucBlue Live. The arrows in the merge channel point to representative cells that appear to be undergoing morphological changes, or to cell debris. (**B**) Quantification of TMRE fluorescence intensity after treatment with E:C at different time points. A minimum of three cells/field and three fields/slide were imaged for each plate at 20× magnification using a Zeiss confocal microscope. The images were analyzed using Image J software, and the change in mean fluorescence among groups was plotted. Normality of distribution of the data was determined using the D-Agostino Omnibus test. A one-way ANOVA analysis was performed followed by a Dunn’s post hoc test using GraphPad Prism software to identify statistical difference among the samples. *N* = 3 for each condition; *N* represents one glass-bottomed 35 mm petri dish. * *p* < 0.05 vs control or T0.

To assess the effect of E:C treatment on mitochondrial membrane potential in whole cells, CV-1 cells were treated with 70 μM E:C in the presence of TMRE. The data presented in Figure 3 demonstrates that after 20 min of treatment with E:C, CV-1 cells exhibited a significant decrease mitochondrial membrane potential, as indicated by the decrease in TMRE uptake compared to the control plate. This decrease was sustained at both the 30- and 45-min time points. Arrows in the merge channel images for both TMRE and NucBlue staining point to representative cells that appear to be undergoing changes in nuclear and/or cytoplasmic morphology or cell debris, and suggest the possibility of a loss of membrane integrity or dying/dead cells. By 60 min, the mitochondrial membrane potential in E:C treated cells appeared completely dissipated. When quantified, the level of TMRE fluorescence in E:C treated cells at the 60-min time point was found to be comparable to that induced by FCCP, a proton ionophore and uncoupler of oxidative phosphorylation.

**Figure 4:**
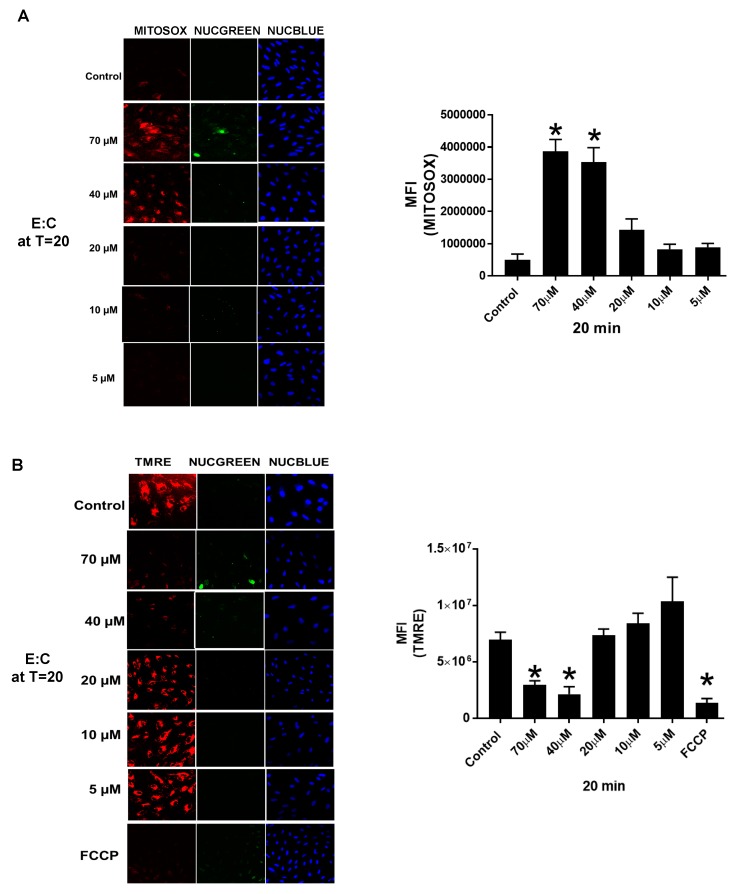
The relationship between E:C-induced superoxide production, membrane potential effects, and cell viability. (**A**) Confocal images and fluorescence intensity measurements in CV-1 Cells treated for 20 min with various concentrations of E:C after staining with the mitochondria-specific superoxide indicator MitoSOX, the cell viability stain NucGreen Dead, and the nuclear stain NucBlue Live. (**B**) Confocal images and fluorescence intensity measurements in CV-1 Cells treated for 20 min with various concentrations of E:C after staining with the mitochondrial membrane potential probe TMRE, the cell viability stain NucGreen Dead, and the nuclear stain NucBlue Live. The proton ionophore and mitochondrial uncoupler, FCCP, was included as a positive control. A minimum of 3 cells/field and 3 fields/slide were imaged for each plate at 20× magnification using a Zeiss confocal microscope. The images were analyzed using Image J software and the change in mean fluorescence among groups was plotted. Normality of distribution of the data was determined using the D-Agostino Omnibus test. A one-way ANOVA analysis was performed followed by a Dunn’s post hoc test using GraphPad Prism software to identify statistical difference among the samples. *N* = 3 for each condition; *N* represents one glass bottomed 35 mm petri dish. * *p* < 0.05 vs control or T0.

To assess the relationship between E:C-induced superoxide generation and loss of mitochondrial membrane potential in whole cells, CV-1 cells were treated with varying concentrations of E:C (5–70 μM) for a constant exposure time (20 min) in the presence of MitoSOX or TMRE. The data presented in Figure 4 indicate that significant changes in ROS generation and mitochondrial membrane potential occurred only at the 40 and 70 μM E:C concentrations, and not at the lower concentrations tested. In order to determine the effect of E:C on cell viability under these conditions, CV-1 cells were co-stained with NucGreen Dead, a nucleic acid stain that emits green fluorescence when bound to the DNA of dead cells. As shown in Figure 4, some evidence cell death under these conditions can be observed only at the highest concentration of E:C tested (70 μM). These results demonstrate that the E:C-induced increase in ROS generation and loss of mitochondrial membrane potential in whole cells can occur concomitantly at sub-lethal concentrations of E:C, as well as suggest that both events precede cell death.

**Figure 5:**
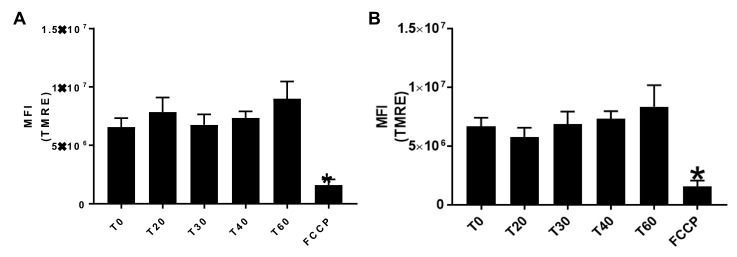
The effect of elesclomol and CuCl_2_, as single agents, on mitochondrial membrane potential in whole cells. Fluorescence intensity measurements of (**A**) elesclomol and (**B**) CuCl_2_ treated CV-cells observed at various time points after staining with the mitochondrial membrane potential probe TMRE. The proton ionophore and mitochondrial uncoupler, FCCP, was included as a positive control. A minimum of three cells/field and three fields/slide were imaged for each plate at 20× magnification using a Zeiss confocal microscope. The images were analyzed using Image J software, and the change in mean fluorescence among groups was plotted. Normality of distribution of the data was determined using the D-Agostino Omnibus test. A one-way ANOVA analysis was performed followed by a Dunn’s post hoc test using GraphPad Prism software to identify statistical difference among the samples. *N* = 3 for each condition; *N* represents one glass-bottomed 35 mm petri dish. * *p* < 0.05 vs control or T0.

Additionally, Figure 5A,B show the data obtained from control experiments in which CV-1 cells were treated up to 1 h with either 70 μM elesclomol or 70 μM copper chloride in the presence of TMRE, NucGreen, and NucBlue stains. The results show that as a single agent, neither compound has any observable effect on mitochondrial membrane potential or cell viability. 

### 3.2. Polarographic Measurement of Oxygen Consumption 

**Figure 6:**
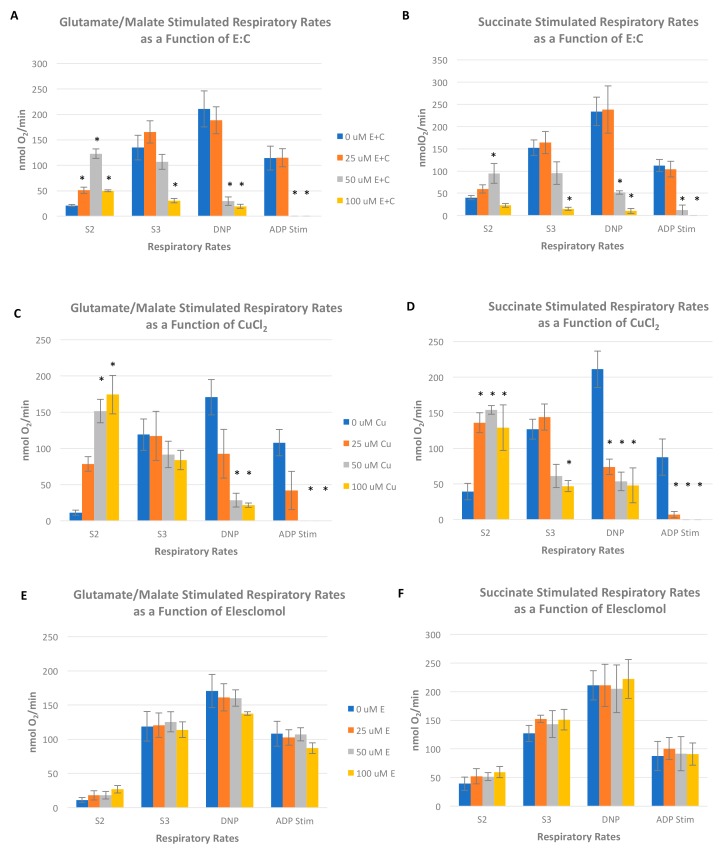
Polarographic measurement of oxygen consumption. Measurements of respiratory rates in intact, isolated rat liver mitochondria were made in the absence or presence of: varying concentrations of the E:C chelate with either (**A**) glutamate/malate or (**B**) succinate as the respiratory substrate; varying concentrations of CuCl_2_ as a single agent with either (**C**) glutamate/malate or (**D**) succinate as the respiratory substrate; or varying concentrations of elesclomol as a single agent with either (**E**) glutamate/malate or (**F**) succinate as the respiratory substrate. Data are expressed as mean values ± SE for at least three independent experiments. A one-way ANOVA was performed followed by a Tukey’s multiple comparison test using GraphPad Prism software; * *p* < 0.05 vs control.

Measurements of oxygen consumption in intact, isolated rat liver mitochondria were made in the absence or presence of varying concentrations of E:C and presented in Figure 6A,B. The initial or state 2 (S2) respiratory rate, in the presence of oxidizable substrate but absence of externally added ADP, is a measure of the basal rate of O_2_ consumption. The results show that when either glutamate/malate (a complex I electron donor) or succinate (a complex II electron donor) was used as the respiratory substrate, E:C stimulated the S2 respiratory rates in a dose dependent manner over a concentration range from 0–50 μM. These data suggest that E:C acts as an uncoupler of oxidative phosphorylation. However, when either glutamate/malate or succinate was used as the respiratory substrate, a significant decrease in O_2_ consumption was observed at the highest concentration of E:C tested (100 μM).

In the presence of ADP, the oxygen consumption rate measures oxidative phosphorylation; that is, the respiratory rate in which substrate oxidation (via electron transfer) is coupled to ATP synthesis. The state 3 (S3) respiratory rate is that which is measured in the presence of substrate and added ADP, while the ADP-stimulated rate is calculated by subtracting the S2 respiratory rate (substrate only) from the S3 rate. The results show that when either glutamate/malate or succinate was used as the respiratory substrate, 25 μM E:C had no significant effect on S3 or ADP-stimulated respiratory rates (Figure 6A,B). However, with either respiratory substrate, at the higher concentrations of E:C tested (50 and 100 μM), the S3 rate was inhibited in a dose-dependent manner and the ADP-stimulated rate was inhibited completely. These data suggest that the higher concentrations of E:C inhibit electron transfer activity in isolated rat liver mitochondria.

The uncoupler-stimulated respiratory rate is induced by addition of the proton ionophore 2,4-dinitrophenol (2,4 DNP) and is essentially a measure of overall electron transfer activity that is no longer coupled to ATP synthesis. Results in Figure 6A,B show that when either glutamate/malate or succinate was used as the respiratory substrate, 25 μM E:C had no significant effect on the DNP-stimulated respiratory rate. However, at the higher concentrations of E:C tested (50 and 100 μM), DNP-stimulated respiratory rates were inhibited in a dose-dependent manner. This decrease in the DNP-stimulated respiratory rate by higher concentrations of E:C is likely a result of membrane disruption due to the compound’s uncoupling effect and/or its generalized inhibition of electron transport activity. 

Polarographic measurement of oxygen consumption in intact, isolated rat liver mitochondria was also made in the absence or presence of varying concentrations of either CuCl_2_ or elesclomol as single agents. The results in Figure 6C show that when glutamate/malate was used as the respiratory substrate, CuCl_2_ acted as an uncoupler of oxidative phosphorylation, inducing a dose-dependent increase in S2 respiratory activity over the range of concentrations tested (25–100 μM E:C), but having no significant effect on the S3 respiratory rate. The observed decrease in the ADP-stimulated (S3-S2) rate therefore occurred as a consequence of (and in proportion to) the increase in the S2 rate, and not as a consequence of inhibition of ATP synthesis and/or electron transport activity. A dose dependent inhibition of the DNP-stimulated respiratory rate was also observed over the range of concentrations tested (25–100 μM). When succinate was used as the respiratory substrate, the uncoupling effect by CuCl_2_ was more immediate and pronounced (Figure 6D). Maximal stimulation of the S2 respiratory rate achieved at 25 μM CuCl_2_ and maintained up to the highest concentration tested (100 μM). The S3 respiratory rate was not affected at a concentration of 25 μM CuCl_2_, but was inhibited by approximately 60% at both the 50 and 100 μM CuCl_2_ concentrations. The DNP-stimulated respiratory rate was inhibited approximately 75% compared to the control value at each concentration of E:C tested (25–100 μM E:C). This decrease in both the S3 and DNP-stimulated respiratory rates is likely due to either a general membrane disruption of the ETC activities, or to an excess of uncoupling agents (CuCl_2_, or CuCl_2_ plus 2,4-DNP) rather than to a site-specific inhibition of electron transport activity. 

Over the range of concentrations tested (25–100 μM) elesclomol as a single agent (in the absence of CuCl_2_) had no significant effect on S2, S3 ADP-stimulated, nor DNP-stimulated rates when either glutamate/malate or succinate was used as the respiratory substrate (Figure 6E,F). 

### 3.3. Enzyme Assays

The effect of varying concentrations of E:C on electron transfer enzyme activities was investigated using freeze-thawed preparations of isolated mitochondria. In order to discern any potential site-specific action of the E:C chelate, assays were chosen such that only limited segments of the respiratory chain were engaged in electron transfer activity at any one time. For example, NADH-ubiquinone oxidoreductase activity is a measure of the rate of electron transfer from the oxidizable substrate NADH, through complex I; succinate-cytochrome c reductase activity is a measure of electron transfer from an oxidizable FADH_2_-linked substrate (succinate), through complex II and III, to cytochrome c; and, cytochrome c oxidase activity measures the rate of complex IV directly by monitoring its rate of oxidation of reduced cytochrome c. 

**Figure 7:**
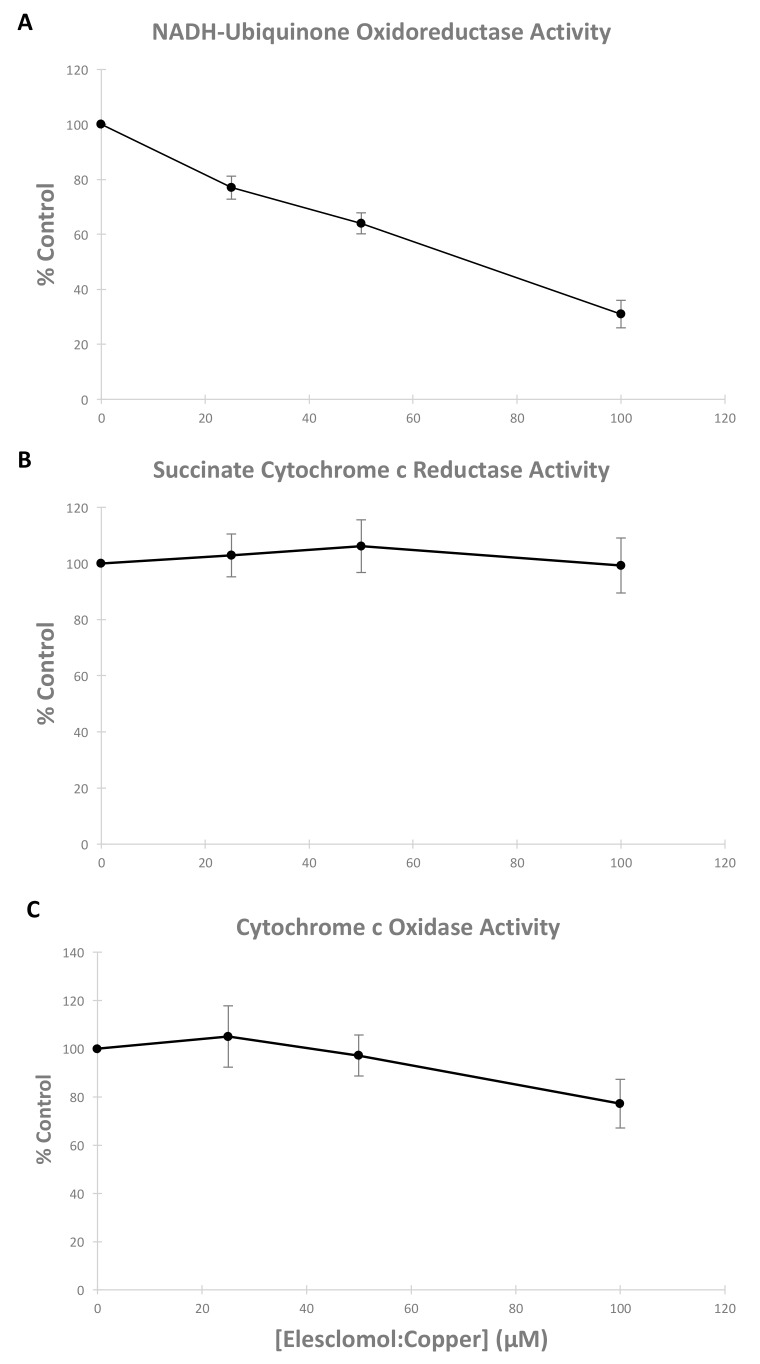
Effect of varying concentrations of E:C on mitochondrial respiratory enzyme complex activity. (**A**) NADH-ubiquinone oxidoreductase activity, (**B**) succinate cytochrome c reductase activity, and (**C**) cytochrome c oxidase activity were measured in freeze-thawed preparations of bovine mitochondria in the absence or presence of varying concentration of E:C. Data presented are the mean of three separate experiments +/− SE.

The effect of E:C on NADH-ubiquinone oxidoreductase activity is shown in Figure 7A. The data indicate that E:C induces a dose-dependent inhibition of mitochondrial NADH-ubiquinone oxidoreductase activity at a concentration range between 0 and 100 µM, with a half maximal inhibitory concentration (IC_50_) achieved at a concentration of approximately 70 μM. The effect of E:C on succinate cytochrome c reductase and cytochrome oxidase activities are shown in Figure 7B,C, respectively. The data indicate no significant effect of E:C on these electron transfer enzyme activities.

## 4. Discussion

The results of this study indicate that E:C treatment in non-tumorigenic cells induces a rapid and substantial increase in mitochondrial superoxide levels (Figure 2). This is consistent with data previously shown for a variety of cancer cells types [1,2] Mitochondria are the main intracellular source of ROS in most tissues. It has been estimated that under physiological conditions, 1–2% of the molecular oxygen consumed by respiring cells is converted to ROS molecules as a byproduct of oxidative phosphorylation [13]. The ROS production occurs when a small fraction of reducing equivalents from complex I, II, or III of the mitochondrial ETC leak electrons directly to molecular oxygen, generating the superoxide anion, O_2_^−^•. The mitochondrial enzyme superoxide dismutase converts O_2_^−^• to H_2_O_2_, which can then acquire an additional electron from a reduced transition metal to generate the highly reactive hydroxyl radical, ^.^OH. Under controlled conditions, ROS play an important role as signaling molecules that mediate changes in cell proliferation, differentiation, and gene transcription [14,15]. However, uncontrolled ROS activity leads to oxidative stress, which can damage intracellular protein and lipid components, affect the integrity of biological membranes, damage both nuclear and mitochondrial DNA (mtDNA), and ultimately lead to irreversible damage and cell death [16]. Interestingly, higher baseline levels of ROS in tumor versus normal cells are known to contribute to the development or maintenance of the malignant phenotype, or both, and render cancer cells more vulnerable to irreversible oxidative damage and consequent cell death [17,18]. It has been suggested that compounds, such as elesclomol, that induce oxidative stress can exploit this unique characteristic of cancer cells by increasing ROS levels beyond the threshold of lethality in cancer cells, while leaving normal cells viable [7]). In fact, elesclomol is one among a number of mitochondria-targeted ROS inducers that have recently shown efficacy as potential anticancer agents [19,20].

Additionally, the data presented here show that treatment of non-tumorigenic cells with the same concentration of E:C that induces a rapid and substantial increase in mitochondrial superoxide levels, also dissipates the mitochondrial membrane potential to an extent sufficient to cause changes in cell morphology suggestive of a loss of membrane integrity or dying/dead cells (Figure 3 and Figure 4). Furthermore, results show that E:C acts directly as an uncoupler of oxidative phosphorylation in intact mammalian mitochondria (Figure 6). 

Interestingly, data obtained in this study show that CuCl_2_ as a single agent also acts as an uncoupler mitochondrial oxidative phosphorylation irrespective of whether a complex I or complex II electron donor is used as a respiratory substrate (Figure 6C,D). In addition, CuCl_2_ was shown to inhibit succinate stimulated S3 respiration in isolated mitochondria. These results are in line with an earlier published report, which showed inhibition of mitochondrial respiration, a decrease in mitochondrial membrane potential, and a marked increase in ROS levels in mitochondria exposed to CuCl_2_ [21]. The authors of that study concluded that copper at high doses can induce mitochondrial dysfunction through non-specific effects on the mitochondrial ETC, and that antioxidants can protect mitochondrial function by reducing the formation of free radicals. In another study investigating the effects of heavy metals on mitochondrial permeability transition (MPT) in isolated rat liver mitochondria, micromolar concentrations of Cu^2+^ as a single agent produced a dose-dependent inhibition of uncoupler-stimulated respiration, membrane potential dissipation, and enhanced membrane permeabilization manifested in mitochondrial swelling and activation of basal respiration [22]. It is important to note that the data presented in Figure 6A–D show that copper as a single agent appears to have a more pronounced uncoupling effect on mitochondria than copper that has been chelated to elesclomol. These results indicate that elesclomol binding mitigates the uncoupling effect of copper.

Finally, the results of this study show that E:C acts as a generalized inhibitor of electron transport activity in isolated, intact mammalian mitochondria, and induces a dose-dependent inhibition of mitochondrial NADH-ubiquinone oxidoreductase activity (complex I) in freeze-thawed mitochondrial preparations (Figure 7A). Complex I is the largest respiratory enzyme complex of the mammalian mitochondrial ETC. It serves as the entry point for electrons donated by NADH, a high-energy electron carrier produced via the citric acid cycle and fatty acid oxidation, and transfers those electrons to the mobile carrier, ubiquinone, thus regenerating the NAD^+^ levels in the mitochondrial matrix. This electron transfer at complex I is coupled to the translocation of protons from the mitochondrial matrix to the intermembrane space. Therefore, complex I plays an integral role in generating the electrochemical proton gradient required for the synthesis of ATP from ADP and P_i_. A recent review describes the important role that complex I also plays in redox control and the biosynthesis of macromolecules and nucleic acids necessary for cell proliferation [20]. It is suggested that these complex I-dependent events contribute to tumor formation, resistance to cell death, and metastasis of cancer cells in part by causing an increase in ROS levels [20]. Interestingly, a number of inhibitors of mitochondrial ETC complex I have been shown to increase mitochondrial ROS production and, consequently, to induce cell death via apoptosis [19,20,23,24]. The data presented in this study suggest that the E:C chelate may act similarly. However, additional experiments will be necessary to determine definitively whether E:C inhibits complex I specifically, thus causing an increase in ROS, or whether complex I, which is the most labile of the respiratory complexes, is simply more susceptible to generalized E:C-induced membrane disruption or ROS produced elsewhere in the mitochondria.

It is of interest to consider the relationship between the uncoupling and ROS generating effects shown to be induced by E:C in this study. It is generally accepted that ROS production is dependent upon the mitochondrial transmembrane electric potential difference (ΔΨ_m_), and that uncoupling agents, which lower ΔΨ_m_, will decrease mitochondrial ROS production [25,26]. However, in this study, dissipation of the mitochondrial membrane potential by E:C in whole cells is shown to coincide with an increase in mitochondrial specific superoxide production (Figure 4). Interestingly, another uncoupler of oxidative phosphorylation, the proton ionophore FCCP, has also been shown to increase mitochondrial ROS production under certain conditions. In one study, exposure of isolated cardiomyocytes to FCCP caused a significant increase in the rate of ROS production compared to control cells, and this increase was shown to be completely blocked by pre-treatment with antioxidants [27]. The authors proposed that the increase in ROS occurred independently of ΔΨ_m_, and raised the possibility that FCCP induced ROS production may be from a source other than the mitochondria. In another study using rat liver mitochondria, the results provided evidence that FCCP caused a burst of H_2_O_2_ production that preceded membrane permeabilization [28]. The results presented in this report showing that E:C both dissipates the mitochondrial membrane potential and causes an increase in mitochondrial specific ROS more likely suggest an uncoupling mechanism involving MPT, which is known to be induced by elevated mitochondrial ROS [29]. Indeed, it has also been shown that low triggering amounts of ROS (possibly generated from mitochondrial ETC inhibition) can cause membrane potential depolarization and a subsequent burst in ROS production, a phenomenon referred to as “ROS-induced ROS release” (RIRR) [30]. Thus, in one possible scenario, inhibition of mitochondrial respiration by E:C (at complex I or non-specifically) may be sufficient to induce triggering amounts of ROS followed by MPT and a consequent burst of ROS. It is also well known that, if not reversed, MPT can lead to structural breakdown of the mitochondrial matrix accompanied by outer mitochondrial membrane rupture and cell death [29]. Of course, the possibility also exists that the different biological effects demonstrated by E:C in this study (i.e., ETC inhibition, uncoupling, and ROS induction) may be independent of one another.

Earlier reports have clearly established the ability of elesclomol to generate high levels of mitochondrial ROS and ultimately induce cell death in a variety of cancer cell lines. Several recent studies show that elesclomol can produce a number of other biological effects in different models, suggesting the possibility of multiple mechanisms of action and cellular targets for the compound. For example, it has been shown that low nanomolar concentrations of elesclomol can be used to shuttle sub-lethal concentrations of copper into the mitochondria as a therapeutic strategy to restore mitochondrial respiratory activity in copper deficient yeast mutants [31]. Using murine, human and zebrafish models, the authors also established broad applicability of elesclomol as a potential therapeutic agent to treat disorders of mitochondrial and cellular copper metabolism. In another study, elesclomol was shown to be a relatively potent bactericidal against a laboratory strain and multidrug resistant clinical isolates of *Mycobacterium tuberculosis* (*Mtb*), and to display additive interactions with known tuberculosis drugs, such as isoniazid and ethambutol, and a synergistic interaction with rifampicin [32]. Interestingly, elesclomol exhibited a >65-fold increase in activity against *Mtb* in the presence of copper compared to non-copper supplemented growth medium. The authors suggest that the ability of elesclomol to enhance ROS generation and accelerate uptake and intracellular accumulation of copper, which is toxic to *Mtb* at high concentrations, as possible mechanisms for the compound’s bactericidal efficacy. Additionally, a very recent report elucidated a unique mechanism of action of cytotoxicity by elesclomol in cancer cells that exhibit proteasome inhibitor resistance or a forced shift away from glycolysis and toward a state of enhanced mitochondrial metabolism, or both [33]. In these cells, there is an upregulation of the activity of ferredoxin 1 protein (FDX1), which is a critical enzyme component in the Fe–S cluster assembly pathway. Results of the study show that E:C binding to FDX1 interferes with the enzyme’s ability to reduce its natural substrate, thereby inhibiting Fe–S cluster formation and resulting in a unique copper-dependent cell death. The authors suggest that other copper ionophores, such as disulfiram, may promote cancer cell death through a similar mechanism. 

The results of the study presented herein provide important information about the mechanisms of mitochondrial and cellular toxicity induced by E:C. First, they reveal at least two additional E:C targets directly affecting mitochondrial bioenergetic function. The data indicate that E:C treatment in non-tumorigenic cells can induce a rapid and substantial increase in mitochondrial superoxide levels and dissipate the mitochondrial membrane potential. Furthermore, the data demonstrate that over the range of concentrations tested, E:C acts as both an uncoupler of oxidative phosphorylation and a generalized inhibitor of electron transport activity in isolated, intact mammalian mitochondria, and induces a dose-dependent inhibition of mitochondrial NADH-ubiquinone oxidoreductase activity in freeze-thawed mitochondrial preparations. These results suggest the possibility that the increase in ROS production and cytotoxicity induced by E:C may at least in part be a result of the uncoupling of mitochondrial oxidative phosphorylation or inhibition of electron transport activity, or both. The results also suggest the possibility that differences between the mitochondria of normal versus cancer cells (e.g., in membrane permeability, response to uncoupling, and complex I structure and function) might somehow contribute to the selective cytotoxicity exhibited by elesclomol for certain cancer cell types. Indeed, nearly a century of scientific research has revealed a number of notable differences in the structure and function of mitochondria between normal and cancer cells, including differences in mtDNA sequence, molecular composition, and metabolic activity (for review see [34]). Additional studies exploring the possibility that differences at the bioenergetic level may increase the vulnerability of cancer cells to elesclomol is warranted and will ultimately contribute to a better understanding of the therapeutic potential of elesclomol as an anticancer compound.
